# Prediction of poorly differentiated hepatocellular carcinoma using contrast computed tomography

**DOI:** 10.1186/1470-7330-14-7

**Published:** 2014-04-22

**Authors:** Kenichiro Nakachi, Hideyuki Tamai, Yoshiyuki Mori, Naoki Shingaki, Kosaku Moribata, Hisanobu Deguchi, Kazuki Ueda, Izumi Inoue, Takao Maekita, Mikitaka Iguchi, Jun Kato, Masao Ichinose

**Affiliations:** 1 Second Department of Internal Medicine, Wakayama Medical University, 811-1 Kimiidera, Wakayama 641-0012, Japan

**Keywords:** Contrast computed tomography, Histological differentiation, Poorly differentiated hepatocellular carcinoma

## Abstract

**Background:**

Percutaneous radiofrequency ablation (RFA) is a well-established local treatment for small hepatocellular carcinoma (HCC). However, poor differentiation is a risk factor for tumor seeding or intrahepatic dissemination after RFA for HCC. The present study aimed to develop a method for predicting poorly differentiated HCC using contrast computed tomography (CT) for safe and effective RFA.

**Methods:**

Of HCCs diagnosed histologically, 223 patients with 226 HCCs showing tumor enhancement on contrast CT were analyzed. The tumor enhancement pattern was classified into two categories, with and without non-enhanced areas, and tumor stain that disappeared during the venous or equilibrium phase with the tumor becoming hypodense was categorized as positive for washout.

**Results:**

The 226 HCCs were evaluated as well differentiated (w-) in 56, moderately differentiated (m-) in 137, and poorly differentiated (p-) in 33. The proportions of small HCCs (3 cm or less) in w-HCCs, m-HCCs, and p-HCCs were 86% (48/56), 59% (81/137), and 48% (16/33), respectively. The percentage with heterogeneous enhancement in all HCCs was 13% in w-HCCs, 29% in m-HCCs, and 85% in p-HCCs. The percentage with tumor stain washout in the venous phase was 29% in w-HCCs, 63% in m-HCCs, and 94% in p-HCCs. The percentage with heterogeneous enhancement in small HCCs was 10% in w-HCCs, 10% in m-HCCs, and 75% in p-HCCs. The percentage with tumor stain washout in the venous phase in small HCCs was 23% in w-HCCs, 58% in m-HCCs, and 100% in p-HCCs. Significant correlations were seen for each factor (p < 0.001 each). Sensitivity, specificity, positive predictive value, negative predictive value, and accuracy for prediction of poor differentiation in small HCCs by tumor enhancement with non-enhanced areas were 75%, 90%, 48%, 97%, and 88%, respectively; for tumor stain washout in the venous phase, these were 100%, 55%, 22%, 100%, and 60%, respectively.

**Conclusions:**

Tumor enhancement patterns were associated with poor histological differentiation even in small HCCs. Tumor enhancement with non-enhanced areas was valuable for predicting poorly differentiated HCC.

## Background

Percutaneous radiofrequency ablation (RFA) is a well-established local treatment for unresectable small hepatocellular carcinoma (HCC), which is a repeatable and safe procedure. Currently, RFA is considered the standard of care for patients with Barcelona-Clinic Liver Cancer (BCLC) 0-A tumors not suitable for surgery [1]. Recently, Forner et al. [2] proposed RFA instead of resection in patients with very early (<2 cm) HCC.

However, several investigators have reported the risk of seeding [3-5], intrahepatic dissemination [6,7], and aggressive recurrence after RFA [8-10]. Some investigators reported that these critical recurrences were related to poor differentiation [4,7]. Therefore, poor differentiation would be a risk factor for tumor seeding or intrahepatic dissemination after RFA for HCC. Furthermore, the prognosis of patients with poorly differentiated HCCs is worse even with radical therapy [11-13]. Along with de-differentiation from well to moderately/poorly differentiated HCC, even small HCCs have a greater tendency for vascular invasion and intrahepatic metastasis [14,15]. Fukuda et al. [16] recommended that, when hepatic function is well preserved, hepatic resection should be the first choice for local control, especially in cases of moderately to poorly differentiated HCC. Therefore, the prediction of poorly differentiated HCC before therapy is crucial for deciding the optimal therapeutic strategy and for safe and effective RFA even for small HCCs.

Contrast computed tomography (CT) is commonly used for definite diagnosis of HCCs on imaging [17]. However, the differential diagnosis of poorly differentiated HCC using contrast CT has not been sufficiently established. In the present study, correlations between the enhancement pattern on contrast CT and histological differentiation, and the ability to predict poorly differentiated HCC using contrast CT were analyzed.

## Results

### Correlation of tumor size and histological differentiation

The histological classification was w-HCC in 56, m-HCC in 137, and p-HCC in 33. Mean diameter by histological classification was 26 ± 13 mm in w-HCCs, 33 ± 20 mm in m-HCCs, and 44 ± 33 mm in p-HCCs. The tumor size was significantly larger as the histological differentiation grade advanced (p = 0.03). In pairwise comparisons, tumor size was significantly smaller for w-HCCs than for m-HCCs and p-HCCs (P = 0.003 and p = 0.001). However, there was no significant difference between m-HCCs and p-HCCs (p = 1.000). The proportions of small HCCs (3 cm or less) in w-HCCs, m-HCCs, and p-HCCs were 86% (48/56), 59% (81/137), and 48% (16/33), respectively. The proportions of w-HCCs, m-HCCs, and p-HCCs in small HCCs were 33% (48/145), 56% (81/145), and 11% (16/145), respectively.

### Correlation between tumor enhancement patterns and histological differentiation

The correlation between tumor enhancement patterns in the arterial phase and histological differentiation is shown in Table 1. The percentage of tumors with tumor stain with non-enhanced areas was significantly higher as the histological differentiation grade advanced (p < 0.001). In pairwise comparisons, there was a significant difference between m-HCCs and p-HCCs. However, there was no significant difference between w-HCCs and m-HCCs. As in all HCCs, there was also a significant correlation even in small HCCs (3 cm or less in diameter).

**Table 1:** Correlation between tumor enhancement pattern in the arterial phase and histological differentiation

**Tumor enhancement in the arterial phase**	**Histological differentiation**			
	**Well**	**Moderately**	**Poorly**	**p-value**
All HCCs	n = 56	n = 137	n = 33	
With non-enhanced areas (n = 74)	7 (13%)	39 (29%)	28 (85%)	
Without non-enhanced areas (n = 152)	49 (88%)	98 (72%)	5 (15%)	<0.001
Small HCCs (3 cm or less)	n = 48	n = 81	n = 16	
With non-enhanced areas (n = 25)	5 (10%)	8 (10%)	12 (75%)	
Without non-enhanced areas (n = 120)	43 (90%)	73 (90%)	4 (25%)	<0.001

HCC, hepatocellular carcinoma.

The p-value was calculated among each differentiation group by with versus without non-enhanced area using Fisher’s exact test.

### Correlation between tumor stain washout and histological differentiation

The correlation between tumor stain washout in the venous phase and histological differentiation is shown in Table 2. The percentage of tumors with tumor stain washout in the venous phase was significantly higher as the histological differentiation grade advanced (p < 0.001). In pairwise comparisons, there were significant differences among all groups (p < 0.05). As in all HCCs, there were also significant correlations even in small HCCs.

**Table 2:** Correlation between tumor stain washout in the venous phase and histological differentiation

**Tumor stain washout in the venous phase**	**Histological differentiation**			
	**Well**	**Moderately**	**Poorly**	**p-value**
All HCCs	n = 56	n = 137	n = 33	
Positive (n = 133)	16 (29%)	86 (63%)	31 (94%)	
Negative (n = 93)	40 (71%)	51 (37%)	2 (6%)	<0.001
Small HCCs (3 cm or less)	n = 48	n = 81	n = 16	
Positive (n = 74)	11 (23%)	47 (58%)	16 (100%)	
Negative (n = 71)	37 (77%)	34 (42%)	0 (0%)	<0.001

HCC, hepatocellular carcinoma.

The p-value was calculated among each differentiation group by positive versus negative tumor stain washout using Fisher’s exact test.

The correlation between tumor stain washout in the equilibrium phase and histological differentiation is shown in Table 3. The percentage of tumors with tumor stain washout in the equilibrium phase was higher as the histological differentiation grade advanced (p < 0.001). However, in comparisons of each pair, no significant difference was observed between m-HCCs and p-HCCs. As in all HCCs, there were also significant correlations even in small HCCs between tumor stain washout in the equilibrium phase and histological differentiation.

**Table 3:** Correlation between tumor stain washout in the equilibrium phase and histological differentiation

**Tumor stain washout in the equilibrium phase**	**Histological differentiation**			
	**Well**	**Moderately**	**Poorly**	**p-value**
All HCCs	n = 56	n = 137	n = 33	
Positive (n = 197)	39 (70%)	125 (91%)	33 (100%)	
Negative (n = 29)	17 (30%)	12 (9%)	0 (0%)	<0.001
Small HCCs (3 cm or less)	n = 48	n = 81	n = 16	
Positive (n = 118)	32 (67%)	70 (86%)	16 (100%)	
Negative (n = 27)	16 (33%)	11 (14%)	0 (0%)	0.003

HCC, hepatocellular carcinoma.

The p-value was calculated among each differentiation group by positive versus negative tumor stain washout using Fisher’s exact test.

### Sensitivity, specificity, positive predictive value, negative predictive value, and accuracy for the prediction of poorly differentiated HCC

Sensitivity, specificity, PPV, NPV, and accuracy for the prediction of p-HCC by each CT finding are shown in Table 4. Sensitivity for p-HCC was inferior by tumor enhancement with non-enhanced areas than by tumor stain washout in the venous phase. However, specificity and accuracy for p-HCC were superior by tumor enhancement with non-enhanced areas than by tumor stain washout in the venous phase. These findings were seen even in small HCCs. Although accuracies for p-HCC in both all HCCs and small HCCs were slightly improved by the combination of tumor enhancement with non-enhanced areas and tumor stain washout in the venous phase, these improvements were not significant.

**Table 4:** Sensitivity, specificity, positive predictive value, negative predictive value, and accuracy of prediction for poorly differentiated hepatocellular carcinoma using CT findings

**CT findings**	**Sensitivity**	**Specificity**	**PPV**	**NPV**	**Accuracy**
All HCCs					
Enhancement with non-enhanced area	85%	76%	34%	97%	77%
Tumor stain washout in the venous phase	94%	47%	23%	98%	54%
Both findings positive	79%	80%	40%	96%	80%
Small HCCs (3 cm or less)					
Enhancement with non-enhanced area	75%	90%	48%	97%	88%
Tumor stain washout in the venous phase	100%	55%	22%	100%	60%
Both findings positive	75%	92%	55%	97%	90%

CT, computed tomography; PPV, positive predictive value; NPV, negative predictive value; HCC, hepatocellular carcinoma.

## Discussion

With respect to a hemodynamic change from m-HCC to p-HCC, Asayama et al. [18] reported that the arterial blood supply decreases significantly. Furthermore, it was also found that, although hypervascular tumor was predominant in p-HCCs, the proportion of hypovascular tumors was higher in w-HCCs and p-HCCs than in m-HCCs on contrast ultrasonography [19] and contrast CT [20]. However, Jang et al. [19] indicated that there was no significant difference in arterial vascularity between w-HCCs and p-HCCs on contrast ultrasonography. Lee et al. [20] also demonstrated that no significant difference was seen in the prevalence of atypical arterial enhancement such as hypoattenuation between w-HCCs and p-HCCs on contrast CT. These studies did not analyze the diagnostic values for p-HCC using arterial hypovascularity. Sanda et al. [21] demonstrated that even small HCCs (diameter up to 2 cm) intermingled with hypovascular areas and hypervascular areas on the arterial phase of contrast CT showed contiguous multinodular type and included p-HCC components. Kawamura et al. [22] reported that heterogeneous enhancement with irregular ring-like structures in the arterial phase of contrast CT is a significant independent predictor of p-HCC. Of course, their heterogeneous enhancement pattern with irregular ring-like structures was included in the criteria of tumor enhancement with non-enhanced areas in the present study. From the above, it is assumed that a hemodynamic change from hypervascularity to hypovascularity in overt HCC means that p-HCC components have been generated in the HCC. Accordingly, the present arterial tumor enhancement classification with or without non-enhanced areas is reasonable for predicting hypervascular HCC including p-HCC components.

With respect to other enhancement pattern findings of contrast CT associated with p-HCC, tumor stain washout in the venous phase has been reported. Nishie et al. [23] indicated that p-HCCs are considered to show faster tumor stain washout on contrast CT than non-p-HCCs. On contrast magnetic resonance imaging, it has also been reported that tumor stain washout in the venous phase was more frequently seen in p-HCCs [24,25]. Furthermore, on contrast-enhanced ultrasonography, tumor stain washout time was significantly less in p-HCCs [19,26]. From the various above contrast studies and the present results, there is no doubt that tumor stain washout becomes faster as the histological differentiation of HCC advances.

In the present study, the diagnostic accuracy for p-HCC using tumor enhancement with non-enhanced areas in the arterial phase of contrast CT was high even in small HCCs. On the other hand, the accuracy for p-HCC by tumor stain washout in the venous phase of contrast CT was not as high. In the present results, tumor enhancement with non-enhanced areas and tumor stain washout in the venous phase were associated with poor histological differentiation even in small HCCs. However, the improvement of accuracies for p-HCC in both all HCCs and small HCCs by the combination of tumor enhancement with non-enhanced areas and tumor stain washout in the venous phase were slight, and these improvements were not significant. Therefore, tumor enhancement with non-enhanced areas appears to be the most valuable finding on contrast CT in the prediction of poorly differentiated HCC.

The present study had some limitations. First, the present study was retrospective. Furthermore, HCCs with no enhancement in the arterial phase of contrast CT were not evaluated. Therefore, the present results cannot be generalized to HCCs without arterial enhancement on contrast CT. Second, needle biopsy samples were used. The assessment of histological differentiation grade using biopsy samples may not reflect the lowest differentiated component in the tumor. Nevertheless, the present results could suggest that the contrast CT enhancement pattern facilitates assessment of histological malignant potential. Third, this study could not show the correlation between the contrast CT enhancement pattern and the prognosis. Kawamura et al. [27] reported that a heterogeneous enhancement pattern with irregular ringed-like structures on dynamic CT is associated with tumor recurrence after RFA. However, their study was very small. In the future, a large-scale cohort study should be conducted to investigate whether these findings will contribute to predicting outcome after RFA.

## Conclusions

In conclusion, arterial tumor enhancement with non-enhanced areas and tumor stain washout in the venous phase were associated with poor histological differentiation even in small HCCs, and tumor enhancement with non-enhanced areas was the most valuable finding in the prediction of poorly differentiated HCC. For safe and effective RFA for small HCCs, systematic resection should be considered as the treatment of first choice for small HCCs with arterial tumor enhancement with non-enhanced areas, because the prevalence of microscopic vascular invasion or intrahepatic metastasis is quite high in p-HCCs. If unresectable, combinations of RFA with transcatheter arterial chemo-embolization should be considered as alternative treatment strategies. However, further study and analysis are required to determine whether this approach actually helps improve the prognosis of small p-HCCs.

## Methods

### Patients

**Figure 1:**
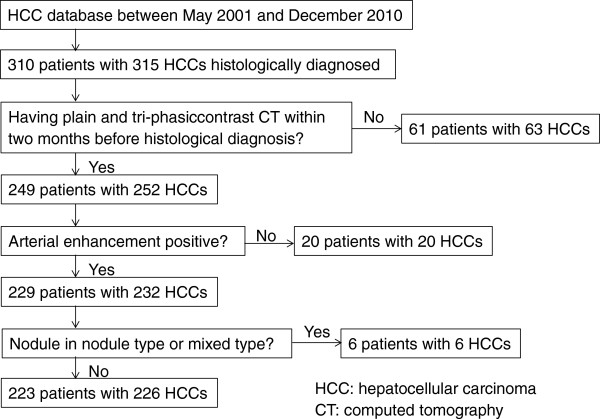
Patient enrollment flowchart.

In our hospital’s HCC database, 310 patients with 315 HCC nodules were histologically diagnosed by tumor biopsy or surgical resection between May 2001 and December 2010. The flowchart of patient enrollment is shown in Figure 1. Of 226 HCC nodules, 165 were diagnosed by tumor biopsy, and 61 were diagnosed by resection. The patients’ characteristics are summarized in Table 5. This retrospective study was approved by our ethics committee and conformed to the Helsinki Declaration. The need for patients to give written, informed consent was waived by our ethics committee.

**Table 5:** Patients’ characteristics

	
Age (years; mean ± SD)	68.3 ± 8.2
Sex (male/female)	147/76
Etiology (HCV/non-HCV)	172/51
Tumor size (mm; mean ± SD)	33 ± 22
Number of tumors (mean ± SD)	1.7 ± 1.2
AFP (ng/mL; mean ± SD)	3026.6 ± 35190.2
AFP-L3 (%; mean ± SD)	14.5 ± 24.7
DCP (mAU/mL; mean ± SD)	4130.0 ± 23805.1
Child class (A/B/C)	161/55/7
Activity stage (A0/1/2/3)	11/99/98/15
Fibrosis grade (F0/1/2/3/4)	7/21/39/68/88

SD, standard deviation; HCV, hepatitis C virus; AFP, alpha-fetoprotein; AFP-L3, lens culinaris agglutinin-reactive alpha-fetoprotein; DCP, Des-gamma-carboxyprothrombin.

### Technique and analysis

All contrast CT examinations were performed with multi-detector row CT scanners having at least 4 detectors (Aquilion, Toshiba Medical Systems, Tochigi, Japan or Light speed VCT, GE Medical Systems, Milwaukee, WI, USA) with a section thickness of 5 mm. In addition to plain images, arterial phase images were obtained 40 seconds after the start of bolus administration. From 2005 onward, the arterial phase was scanned with an automatic bolus-tracking program. Venous and equilibrium phase images were obtained at 70 seconds and 180 seconds, respectively. All patients received a non-ionic iodinated contrast medium at a dose of 580 mgI/kg; it was administered to all patients by an automated power injector for 30 seconds (19.3 mgI/kg/s).

**Figure 2:**
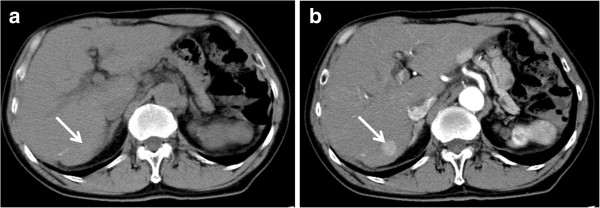
**Tumor enhancement without non-enhanced areas.** The pre-contrast image **(a)** shows an iso-density tumor. In comparison with pre-contrast image, the tumor stain has no non-enhanced areas in the arterial phase **(b)**. The tumor is indicated by arrows.

**Figure 3:**
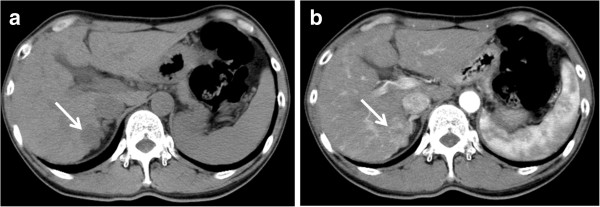
**Tumor enhancement with non-enhanced areas.** The pre-contrast image **(a)** shows a low-density tumor. In comparison with the pre-contrast image, the tumor stain has non-enhanced areas in the arterial phase **(b)**. The tumor is indicated by arrows.

Contrast CT findings related to tumor enhancement pattern and washout were categorized as follows. Tumor enhancement pattern in the arterial phase was classified into two categories, with and without non-enhanced areas (Figures 2 and 3). Tumor stain obtained during the arterial phase that disappeared during the venous or equilibrium phase, with the tumor becoming hypodense, was categorized as positive for washout. Images obtained by contrast CT were independently analyzed using the above criteria of enhancement patterns without reference to histological differentiation by two experienced readers with more than 20 years of experience in liver imaging. Any disagreements in interpretation were resolved by consensus.

Needle biopsies of tumors were performed using an 18-gauge needle (Bard Monopty® C.R. Bard Inc., Covington, GA, USA). Liver biopsy was performed using a 16-gauge needle. Histological findings were classified using the METAVIR scoring system [28]. All biopsy and resected specimens were examined by two experienced pathologists, without reference to the CT findings of their tumors and surrounding livers. According to the International Working Party classification [29], HCC histology was classified into three types: well differentiated (w-), moderately differentiated (m-), and poorly differentiated (p-) HCCs. If heterogeneous differentiation was found in the obtained HCC tissue, differentiation grade was classified based on the lowest differentiated grade. Any discrepancies between the two pathologists with more than 20 years of experience in liver pathology were resolved by discussion to reach consensus.

### Statistical analysis

Values are expressed as means ± standard deviation (SD). The correlation between tumor size and histological differentiation was analyzed using the Jonckheere-Terpstra test. The correlation between the enhancement pattern and histological differentiation was analyzed using Fisher’s exact test or the chi-square test of independence. Sensitivity, specificity, positive predictive value (PPV), negative predictive value (NPV), and accuracy for diagnosis of p-HCC were calculated according to findings on contrast CT. Accuracy between groups was compared using the McNemar test. A p value less than 0.05 was considered significant. All analyses were performed using the SPSS 20.0 software package (SPSS, Inc., Chicago, IL, USA).

## Abbreviations

HCC: Hepatocellular carcinoma; RFA: Percutaneous radiofrequency ablation; CT: Computed tomography; BCLC: Barcelona-clinic liver cancer; w: Well differentiated; m: Moderately differentiated; p: Poorly differentiated; SD: Standard deviation; PPV: positive predictive value; NPV: Negative predictive value.

## Competing interests

The authors declare that they have no competing interests.

## Authors’ contributions

NK, TH and IM designed and proposed the research; all authors approved the analysis and participated in drafting the article; MY, SN, MK, DH, UK, II, MT, IM, and KJ collected the clinical data; TH and IM analyzed imaging examinations; NK and TH performed the statistical analysis; NK and TH wrote the manuscript. All authors read and approved the final manuscript.
